# Draft genome of *Staphylococcus epidermidis* clinical isolate OGSA-Sep-145 from an implant-related spinal infection

**DOI:** 10.1128/mra.00416-25

**Published:** 2025-06-24

**Authors:** Vincenzo Pennone, Matteo Briguglio, Elena De Vecchi, Riccardo Cecchinato, Arianna B. Lovati

**Affiliations:** 1 IRCCS Istituto Ortopedico Galeazzi, Cell and Tissue Engineering Laboratory 46767, Milan, Italy; 2 IRCCS Istituto Ortopedico Galeazzi, Laboratory of Nutritional Sciences 46767, Milan, Italy; 3 IRCCS Istituto Ortopedico Galeazzi, Laboratory of Clinical Chemistry and Microbiology 46767, Milan, Italy; 4 GSpine, IRCCS Istituto Ortopedico Galeazzi 46767, Milan, Italy; 5 Department of Biomedical Sciences for Health, University of Milan 9304, Milan, Italy; Wellesley College, Wellesley, USA

**Keywords:** prosthesis infections, genome analysis

## Abstract

*Staphylococcus epidermidis*, a skin commensal, is a major cause of orthopedic implant-related infections due to its biofilm-forming ability and immune evasion strategies. Here, we present the draft genome of strain OGSA-Sep-145, isolated from a spinal infection, providing insights into antimicrobial resistance and virulence mechanisms in chronic infections.

## ANNOUNCEMENT

Implant-related orthopedic infections represent a major clinical concern due to persistent biofilm-forming pathogens and the rise of antimicrobial resistance (1). *Staphylococcus epidermidis*, a commensal bacterium of human skin, has emerged as a leading cause of implant-related infections. Its ability to form biofilms on abiotic surfaces and evade host immune responses makes it a formidable pathogen in chronic infections (2). Whole-genome analysis of clinical *S. epidermidis* isolates is crucial for elucidating virulence factors, resistance determinants, and strain-level epidemiological features. We report the draft genome of an *S. epidermidis* strain isolated at the IRCCS Ospedale Galeazzi Sant’Ambrogio (45.52234 N 9.09638 E). Following national and institutional guidelines, written informed consent was obtained on 24 June 2024. This study is exempt from Institutional Review Board approval. The patient was a 62-year-old male with chronic spinal implant infection complicated by abscess formation in 2020, after the first arthrodesis surgery in 2018. Strain OGSA-Sep-145 was isolated by swabbing a titanium screw from an infected spinal implant at the L4–L5 level during surgery. Sample processing was carried out under a biosafety cabinet following Good Diagnostic Laboratory Practices, within an ISO 9001-certified laboratory. The strain was recovered from enrichment broths (Brain Heart Infusion and Thioglycollate) after 4 days of incubation at 37°C. Phenotypical identification was performed using the Vitek2 system (BioMerieux Mercy L'Etoile, France) with a Gram Positive (GP) identification card.

For sequencing, a single colony was subcultured at 37°C in 10 mL of Brain Heart Infusion (BHI) broth overnight. Genomic DNA was extracted using the DNeasy PowerSoil Pro Kit (Qiagen, Milan, Italy). Libraries and sequencing were outsourced to Novogene (https://www.novogene.com/amea-en/) using the Next Generation Sequencing (NGS) DNA Library Prep Set kit (Cat No PT004) and Illumina NovaSeq X Plus Series (PE150) with 2 × 150 bp paired-end reads, achieving ~400× coverage. The library was quantified using Qubit and quantitative Polymerase Chain Reaction (qPCR), and fragment sizes were assessed using an Agilent 5400 (Agilent, CA, US). Raw reads were downloaded from the Novogene Customer Service System (CSS) platform and analyzed using PATRIC tools on the Bacterial and Viral Bioinformatics Resource Center (https://www.bv-brc.org/) (3). Reads were trimmed using TrimGalore v0.6.10 (4) and normalized with BBNorm (5), and assembly errors were polished with Bowtie 2 v2.5.4 (6) and corrected with Pilon v1.24 (7). The genome was assembled using Unicycler v0.4.8 (8) with default parameters. Contigs <500 bp were excluded. Assembly quality was assessed with Quast v5.0.2 (9), resulting in 44 contigs with a total genome length of 2,416,621 bp (Table 1). Contamination screening using the NCBI FCS-GX toolset via Galaxy (https://usegalaxy.org/) (10) established the absence of sequencing adaptors and contaminants. Genome annotation was performed with NCBI PGAP v6.10 (11), and taxonomic identification was performed by PATRIC RASTtk (12), revealing 2,245 Coding Sequences (CDS), 48 tRNA, and 4 rRNA genes. A total of 1,851 proteins had functional assignments, including 518 with Kyoto Encyclopedia of Genes and Genomes (KEGG) pathway mapping, while 434 were hypothetical proteins.

**TABLE 1:** Genome features of *S. epidermidis* OGSA-Sep-145

Feature	Count
GenBank accession number	JBNADY000000000
BioProject number	PRJNA1248221
BioSample	SAMN47853561
SRA accession number	SRR33065699
*N* contigs	44
Largest contig	208,828
Total length	2,416,621
GC^*a*^ (%)	31.94
N50	101,596
N90	36,385
L50	8
L90	24
CDS (PGAP)	2,245
tRNA (PGAP and PATRIC)	48
rRNA (PGAP and PATRIC)	4
Hypothetical proteins (PATRIC)	434
Pseudogenes (total)	55
Pseudogenes (ambiguous residues and PATRIC)	0 of 55
Pseudogenes (frameshifted and PATRIC)	41 of 55
Pseudogenes (incomplete and PATRIC)	26 of 55
Pseudogenes (internal stop and PATRIC)	16 of 55
Pseudogenes (multiple problems and PATRIC)	20 of 55
Proteins with functional assignments (PATRIC)	1,851
Proteins with pathway assignments (PATRIC)	518
Antibiotic resistance (PATRIC and CARD)	15
Antibiotic resistance (PATRIC and NDARO)	8
Antibiotic resistance (PATRIC and PATRIC)	40
Drug target (PATRIC and DrugBank)	24
Drug target (PATRIC and TTD)	12
Transporter (PATRIC and TCDB^*a*^)	37
Virulence factor (PATRIC and VFDB^*a*^)	2
Virulence factor (PATRIC and Victors)	19

^*a*^ VFDB, Virulence Factor Database; TCDB, Transporter Classification Database; GC, Guanine Cytosine.

**Fig 1:**
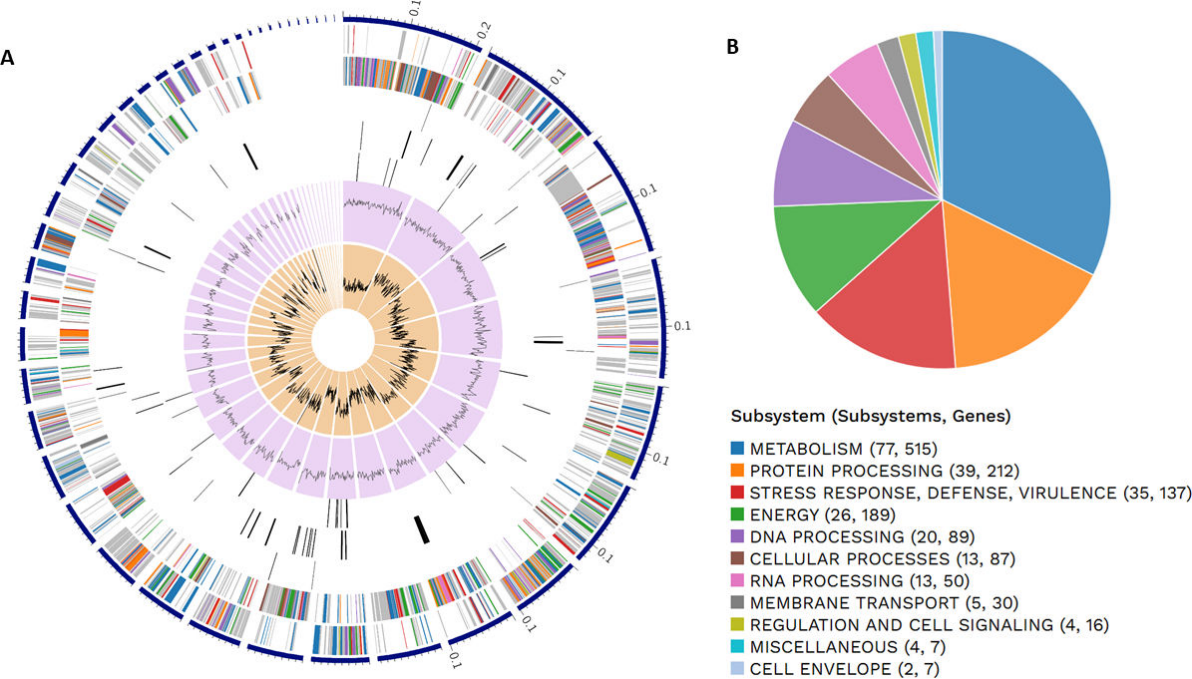
(**A**) Circular graphical display of the distribution of the genome annotations. This includes, from outer to inner rings, the contigs, CDS on the forward strand, CDS on the reverse strand, RNA genes, CDS with homology to known antimicrobial resistance genes, CDS with homology to known virulence factors, GC content, and GC skew. (**B**) An overview of subsystems is shown in the pie chart. The color palette used in both panels **A** and **B** indicates the subsystems to which these genes belong.

Detected genes included known transporters, drug targets, virulence factors, and antibiotic resistance determinants (Table 1). Subsystems and functional annotations are graphically presented in Fig. 1. Default parameters were used unless stated otherwise.

## Data Availability

This Whole Genome Shotgun project has been deposited at DDBJ/ENA/GenBank under accession JBNADY000000000. The version described here is JBNADY010000000 and included in the BioProject number PRJNA1248221 and BioSample SAMN47853561. Raw reads have been deposited in the Sequence Read Archive (SRA) under accession number SRR33065699.
